# Relationship of Body Adiposity with Platelet Function in Obese and Non-obese Individuals

**DOI:** 10.7759/cureus.6815

**Published:** 2020-01-29

**Authors:** Ishfaq A Bukhari, Syed S Habib, Alaa Alnahedh, Futoon Almutairi, Lama Alkahtani, Latefa A Alareek, Ghadah A Assiri

**Affiliations:** 1 Pharmacology, College of Medicine, King Saud University, Riyadh, SAU; 2 Physiology Department, College of Medicine and King Saud University Medical City, King Saud University, Riyadh, SAU; 3 Pharmacology Section, College of Medicine and King Saud University Medical City, King Saud University, Riyadh, SAU; 4 Clinical Pharmacology Department, College of Pharmacy, King Saud University, Riyadh, SAU

**Keywords:** obesity, body composition, adiposity, platelet function

## Abstract

Background

Adiposity is firmly linked to a higher incidence of various cardiovascular and metabolic morbidities, including diabetes, hypertension, and thromboembolism. This research study was aimed to verify the association of increased adiposity and hyperreactivity of platelets in obese and non-obese individuals.

Methods

This cross-sectional study was conducted on 42 subjects aged 18 years and above. Subjects were divided into obese and non-obese groups based on their body mass index (BMI). The data was collected through self-administered questionnaires. All participants underwent body composition analysis. Blood samples were collected from all subjects and taken to the Pharmacology Department for the preparation of platelet-rich plasma (PRP) and poor platelet plasma (PPP). Platelet aggregation was induced by arachidonic acid and was monitored with a Bio/Data multichannel aggregation profiler (Bio/Data Corp., Horsham, PA, USA).

Results

Significant differences were observed in most parameters, such as fat mass, body fat percentage, free fat mass (FFM), the percentage of trunk fat, total body water, waist-hip ratio (WHR), and basal metabolic rate (BMR) of obese and non-obese subjects. The average percent of platelet aggregation in obese and non-obese subjects was 56.33 ± 15.62 and 59.38 ± 12.62, respectively. The average area under the curve (AUC) for platelet aggregation for both groups was 339.33 ± 191.55 and 342 ± 146.68, respectively. Platelet function was not significantly different and didn’t positively correlate with most parameters of the body composition, except WHR, which positively correlated with AUC for platelet function.

Conclusion

There was no significant direct correlation between adiposity and platelet activation in obese subjects. However, a significant positive correlation of AUC for platelet aggregation with WHR was observed (resistance (r)-value: 0.307, p < 0.05). These findings suggest that WHR could be an effective determinant to assess the risk of thromboembolism in obese individuals.

## Introduction

Obesity remains one of the very serious but often underestimated threats to public health. Recent global epidemics have documented a dramatic increase in adult obesity rates throughout the world since the 1980s. It has been stated by World Health Organization (WHO) that nearly 13% of the world’s adult population were obese in 2014, defined as having a body mass index (BMI) equal to 30 or more [1]. The alarming prevalence of obesity has raised public health concerns because of the potentially critical health consequences over the short and long-term [2]. Obesity not only affects body weight homeostasis but also perpetuates and amplifies the metabolic disturbances, leading to a high risk of morbidity and mortality. For instance, adiposity has been firmly linked to a higher incidence of various cardiometabolic morbidities, including diabetes, hypertension, and dyslipidemia, which are considered critical components of thrombotic complications [3]. Moreover, mounting evidence has supported the firm association of adiposity with dyslipidemia, contributing to the excess risk of atherogenesis [4].

Obesity modulates endothelial damage during the earliest phases of atherogenesis by producing bioactive molecules known as adipokines [5-7]. Accumulating evidence has revealed the pivotal mechanistic role of leptin in the development of intravascular thrombosis. Furthermore, It has been proposed that increased levels of leptin significantly impair platelet function [8]. Platelets serve the primary purpose of maintaining normal hemostasis during vessel injury [9]. Once activated, platelets participate in the early steps of atherogenesis by adhesion to the vessel wall following injury and platelet aggregation [10]. Interestingly, previous studies observed platelet hyperaggregability in obese individuals [11-12]. Based on this critical observation, the current study was aimed to establish a rational link between adiposity and the high tendency of platelet hyperreactivity.

To the best of our knowledge, data on the relation of platelet function with body composition remain poorly investigated, and there are many missing links in this area. Therefore, in this study, we explored the association of increased adiposity and platelet hyperaggregability in obese and non-obese adults. Our study aimed to provide useful insights into understanding the role of adiposity in altered platelet function that could be used as an indicator for thromboembolism in obese individuals who have a high risk of cardiovascular events, such as stroke.

## Materials and methods

Study design

This cross-sectional study was comprised of 42 healthy Saudi adults aged 18 years and above. Convenient sampling techniques were used at the Department of Pharmacology and Physiology, College of Medicine, King Khalid University Hospital, Riyadh, Saudi Arabia between the periods of November 2017 to April 2018. The study was approved by the Institutional Ethics Committee, College of Medicine, King Khalid University Hospital, King Saud University, Riyadh. 

Study tool

A total of 51 adults visiting the outpatient clinic were recruited and 42 adults were enrolled in the study. The subjects were further categorized into obese (BMI ≥ 30 kg/m^2^) and non-obese groups [1]. A series of self-administered questionnaires were provided to the participants to collect the demographics, as well as a consent form for the study.

Study protocol

Blood was collected via venipuncture and measurement of body composition analysis was taken. Any participants with a significant medical history of diabetes, hypercholesterolemia, or any other lipid disorder were excluded. Participants with a drug history (particularly of aspirin or nonsteroidal anti-inflammatory drugs (NSAIDs)), smokers, and those diagnosed with diabetes or blood disorders were also excluded.

All participants underwent body composition analysis. Body composition, including body fat %, fat mass, free fat mass (FFM), trunk fat %, trunk fat mass, trunk fat-free mass, predicted muscle mass, total body water, and basal metabolic rate (BMR)), was analyzed via a bioelectrical impedance analysis (BIA) with a commercially available body analyzer (Tanita Corp., Arlington Heights, IL, USA). The subjects were asked to take off their shoes and then stand over the machine’s electrodes and place their hands on the handgrip electrodes pair. Data was recorded in one to two minutes.

Other body composition indices, such as BMI and waist-hip ratio (WHR), were also performed. BMI was calculated via BIA. Waist circumference was measured in inches at the level of the highest point of the iliac crest. Hip circumference was measured at the widest area over the hips in inches. Blood pressure was measured using a sphygmomanometer, and the respiratory rate was recorded for each participant. A blood sample was collected and mixed with a 3.8% (w/v) sodium citrate solution (9:1). A blood sample was centrifuged at 1,000 rpm for four minutes at 20° C to obtain platelet-rich plasma (PRP). Platelet-poor plasma (PPP) was prepared by centrifugation of the remaining blood sample at 3,000 rpm for 15 minutes. Platelet aggregation was studied according to the method as described by Saeed et al. [13]. Aggregation studies were carried out at 37° C. Platelet aggregation was induced by arachidonic acid (0.8 mM) and expressed as percentage inhibition compared to control at five minutes after challenge. Platelet aggregation was monitored with the Bio/Data multichannel aggregation profiler (Bio/Data Corp., Horsham, PA, USA) using 450 μl samples of PRP.

Statistical analysis

The data were expressed as mean ± standard deviation (SD). The student t-test was used for normally distributed data and the Mann-Whitney U test for skewed data. We used the Kolmogorov test to see whether the data followed a normal distribution. Spearman's rank order and Pearson correlations were also determined. A p-value < 0.05 was considered significant.

## Results

A total of 42 subjects were studied and divided into two groups (non-obese and obese) based on a BMI cutoff of 29.9 with a mean age of 32.1 ± 11.192 years and BMI of 34.81 ± 13.391. All subjects were non-smokers and with no drug history, particularly of aspirin or NSAIDs, for the last one week. A family history of subjects for metabolic syndrome was also taken into consideration. Significant differences were observed in most of the parameters, such as fat mass, visceral fat rating (VFR), body fat percentage, FFM, trunk fat, total body water, WHR, BMR, systolic blood pressure (SBP), and diastolic blood pressure (DBP).

**Figure 1:**
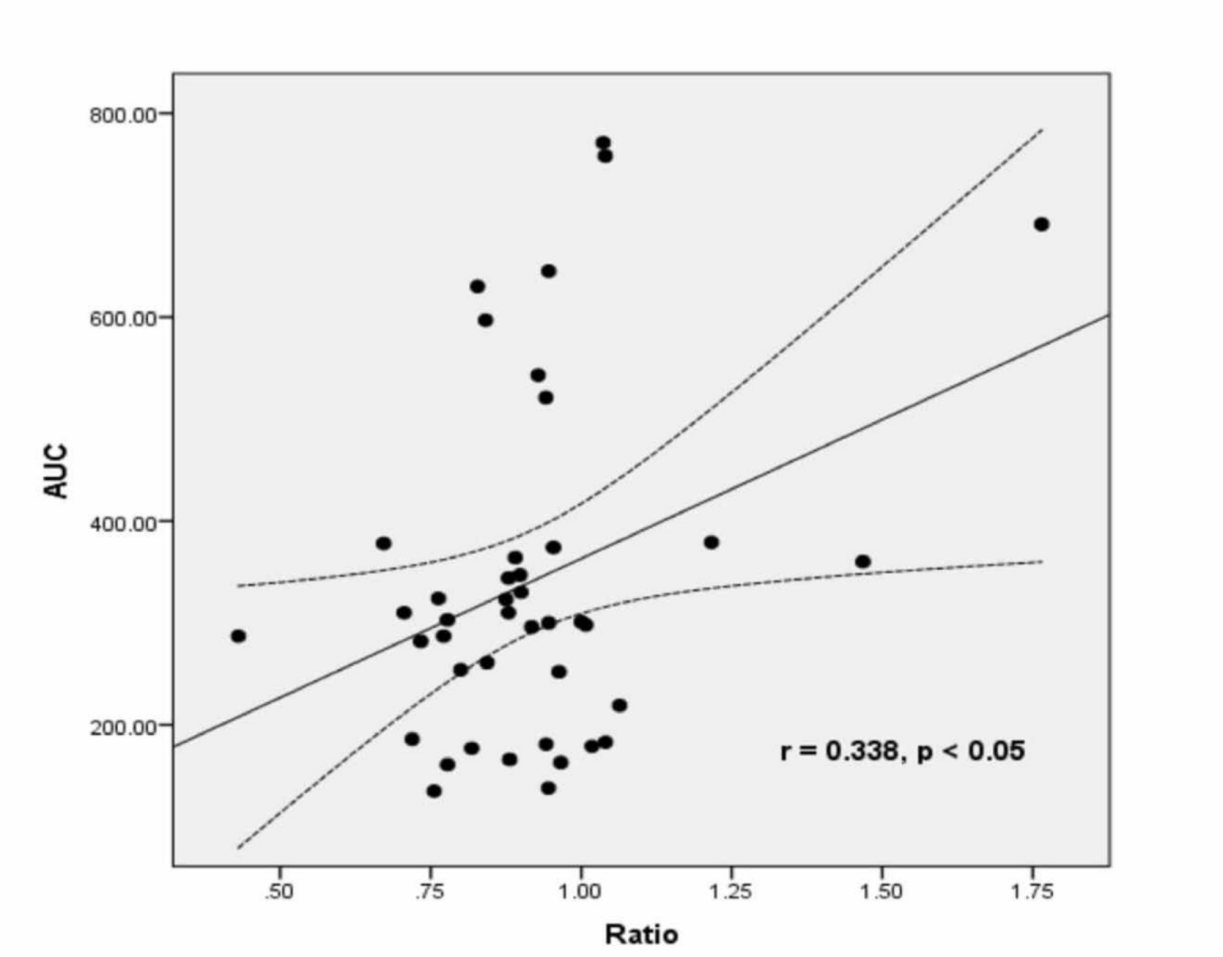
Correlation between WHR and AUC for PA in all subjects showing significant positive correlation (r = 0.338, p < 0.05) AUC: area under the curve; PA: primary aggregation; WHR: waist-hip ratio

**Figure 2:**
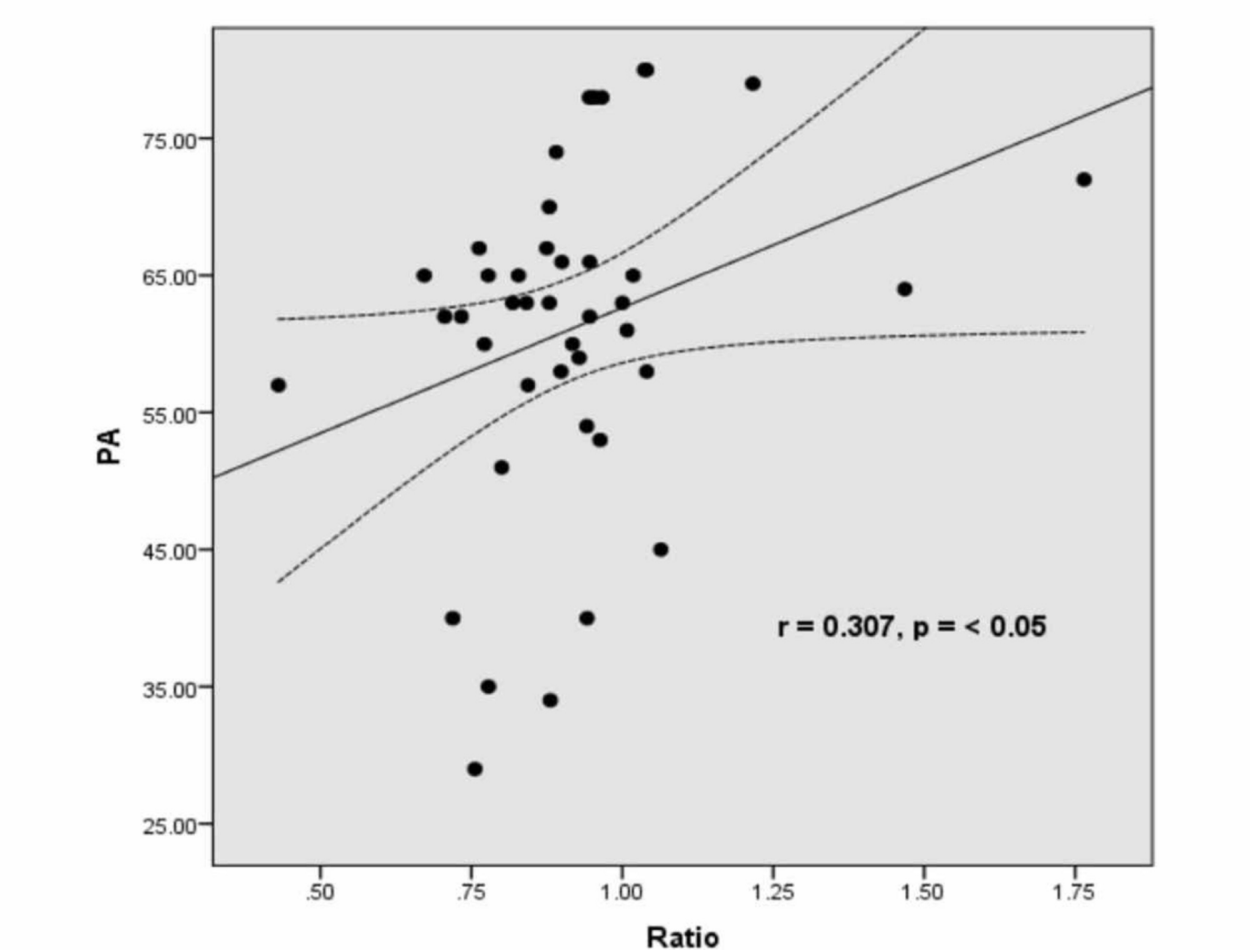
Correlation between WHR and PA in all subjects showing significant positive correlation (r = 0.307, p < 0.05) PA: primary aggregation; WHR: waist-hip ratio

A comparison of demographics and body composition analyses between non-obese and obese groups is shown in Table 1. Physical activity (resistance (r)-value = 0.495, p = 0.001) and BMI (r = 0.332, p = 0.032) correlated negatively with age, while body fat percentage (r = 0.347, p = 0.032), fat mass (r = 0.306, p = 0.049), visceral fat (r = 0.503, p = 0.001), and WHR (r = 0.307, p < 0.05) correlated positively with age. Table 2 shows a comparison of platelet function studies between non-obese and obese subjects. The difference was non-significant for all parameters. However, in Figures 1-2, significant positive correlations were observed between WHR and primary aggregation (PA) (r = 0.307, p < 0.05) and AUC for PA (r = 0.338, p < 0.05), respectively. Tables 3-4 express Spearman correlation analysis between body composition parameters, clinical characteristics, and platelet function studies in all subjects.

**Table 1:** Comparison of Demographic Data and Body Composition Between Obese and Non-obese Groups The comparison was done by Student t-test BMI: body mass index; BMR: basal metabolic rate; DBP: diastolic blood pressure; Desfat: amount or percentage of normal desirable fat content; FFM: free fat mass; SBP: systolic blood pressure; SD: standard deviation; TBW: total body weight; VFR: visceral fat rating; WHR: waist-hip ratio

Variables	Group 1 (non-obese) (mean ± SD)	Group 2 (obese) (mean ± SD)	p-value
Age (years)	29.33 ± 9.446	34.86 ± 12.31	0.111
Height (cm.)	165.76 ± 8.763	163 ± 9.545	0.335
Weight (kg)	61.9 ± 12.653	125.19 ± 19.854	0.000
BMI	22.48 ± 4.02	47.14 ± 5.659	0.000
BMR	5,933.81 ± 954.076	8,992.14 ± 1,707.485	0.000
Fat %	24.38 ± 7.743	45.81 ± 7.16	0.000
Fat Mass (kg)	15.1 ± 7.273	57.25 ± 13.806	0.000
FFM	46.76 ± 8.233	68.9 ± 13.579	0.193
TBW	33.9 ± 5.029	49.33 ± 9.557	0.000
VFR	3.95 ± 3.626	15.57 ± 9.179	0.000
Fat mass	17.6 ± 4.16	24.1 ± 4.745	0.000
Desfat	27.45 ± 6.533	27 ± 6.569	0.153
Whole Body	693.95 ± 69.885	494.38 ± 59.051	0.000
Right Leg	286.1 ± 31.337	181.81 ± 24.602	0.000
Left Leg	286.05 ± 31.57	184.14 ± 27.049	0.000
Right Arm	375.57 ± 50.317	291.24 ± 33.657	0.000
Left Arm	380.67 ± 43.314	291.76 ± 41.416	0.000
Right leg fat%	24.81 ± 10.638	43.76 ± 10.63	0.000
Fat Mass	2.71 ± 1.488	10.24 ± 3.434	0.000
FFM	8.29 ± 1.765	12.9 ± 2.844	0.000
Left Leg Fat %	25.24 ± 11.229	43.48 ± 11.143	0.000
Fat Mass	2.62 ± 1.465	10.05 ± 3.556	0.000
FFM	8.05 ± 1.717	12.86 ± 2.971	0.000
Right Arm Fat %	22.95 ± 7.947	53.29 ± 10.776	0.000
Fat Mass	0.71 ± 0.644	4.43 ± 1.748	0.000
FFM	2.43 ± 0.676	3.71 ± 1.146	0.000
Left Arm Fat %	24.05 ± 8.357	55.71 ± 9.312	0.000
Fat Mass	0.71 ± 0.644	5.05 ± 1.91	0.000
FFM	2.43 ± 0.676	3.86 ± 0.793	0.000
Trunk Fat %	22.62 ± 8.599	44.67 ± 5.407	0.000
Fat Mass	8.95 ± 6.407	27.67 ± 5.323	0.000
FFM	25.9 ± 3.974	34.05 ± 5.554	0.000
Ratio	1 ± 0	1 ± 0.316	0.055
SBP mmHg	126.71 ± 16.876	153.67 ± 26.362	0.000
DBP mmHg	74.71 ± 11.208	88.67 ± 15.602	0.002

**Table 2:** Comparison of Platelets Function Studies Between Non-obese and Obese Subjects Comparison was done by Student t-test AUC: area under the curve; DA: disaggregation; FA: final aggregation; LP: lag phase; MA: maximum aggregation; PA: primary aggregation; PS: primary slope; SD: standard deviation

Variables	Group 1 (non-obese) (mean ± SD)	Group 2 (Obese) (mean ± SD)	P-Value
PA (%) (initial aggregation In the biphasic pattern)	59.38 ± 12.623	56.33 ± 15.618	0.365
PS (%) (initial or only slope)	32.86 ± 6.044	30.76 ± 7.835	0.338
AUC (% per min.)	342 ± 146.687	339.33 ± 191.547	0.960
LP (%) (time between injection of reagent and initiation of aggregation)	2.43 ± 6.193	0.81 ± 3.71	0.310
DA (%) (measurement after maximum aggregation occurs where aggregates break up and disaggregate)	0.14 ± 0.655	1.24 ± 3.33	0.147
MA (%)	59.38 ± 12.623	56.33 ± 15.618	0.491
FA (%)	57.76 ± 12.621	53.67 ± 16.865	0.3781

**Table 3:** Spearman Correlation Analysis Between Body Composition Parameters and Platelets Function Studies Data is expressed as mean ± standard deviation * Correlation was significant at the 0.05 level (2-tailed) ** Correlation was significant at the 0.01 level (2-tailed) AUC: area under the curve (percentage/minute); BMI: body mass index; BMR: basal metabolic rate; DA: disaggregation (measurement after maximum aggregation occurs where the aggregates break up and disaggregate); FA: final aggregation; FFM: free fat mass; LP: Lag Phase (time between injection of the reagent and the initiation of aggregation); MA: maximum aggregation; PA: primary aggregation (initial aggregation in the biphasic pattern); PS: primary slope (initial or only slope); SA: secondary aggregation; SS: secondary slope (secondary wave in the case of biphasic pattern); TBW: total body weight; VFR: visceral fat rating; WHR: waist-hip ratio

	Age	BMI	BMR	Fat %	Fat Mass	FFM	TBW	VFR	WHR	PA	AUC	DA	MA	FA
Age	1													
BMI	0.249	1												
BMR	0.01	.770^**^	1											
Fat %	0.296	.903^**^	.513^**^	1										
Fat Mass	0.071	.412^**^	.426^**^	.329^*^	1									
FFM	0.036	.750^**^	.961^**^	.486^**^	.400^**^	1								
TBW	0.048	.725^**^	.990^**^	.445^**^	.414^**^	.952^**^	1							
VFR	.441^**^	.579^**^	.533^**^	.461^**^	-0.03	.487^**^	.557^**^	1						
WHR	0.196	.333^*^	.415^**^	0.203	0.113	.408^**^	.428^**^	0.159	1					
PA	0.082	0.235	.356^*^	0.098	0.249	.363^*^	.359^*^	0.131	.307^*^	1				
AUC	0.051	0.002	0.091	-0.071	-0.144	0.092	0.103	-0.129	.338^*^	.493^**^	1			
DA	0.114	0.193	0.104	0.184	.419^**^	0.075	0.1	0.097	0.019	-0.108	-0.302	1		
MA	0.086	-0.087	-0.061	-0.055	-0.282	-0.026	-0.068	-0.118	0.205	.643^**^	.692^**^	-.474^**^	1	
FA	0.072	-0.11	-0.084	-0.073	-.322^*^	-0.048	-0.09	-0.125	0.182	.607^**^	.672^**^	-.581^**^	.991^**^	1

**Table 4:** Spearman Correlation Analysis Between Clinical and Demographic with Platelet Function Studies * Correlation was significant at the 0.05 level (2-tailed) ** Correlation was significant at the 0.01 level (2-tailed) AUC: area under the curve (percentage/minute); BMR: basal metabolic rate; DA: disaggregation (measurement after maximum aggregation occurs where the aggregates break up and disaggregate); DBP: diastolic blood pressure; FA: final aggregation; MA: maximum aggregation; PA: primary aggregation (initial aggregation in the biphasic pattern); SBP: systolic blood pressure

	Age	SBP	DBP	BMR	PA	AUC	DA	MA	FA
Age	1								
SBP	.304	1							
DBP	.315^*^	.791^**^	1						
BMR	.010	.577^**^	.529^**^	1					
PA	.082	.280	.167	.356^*^	1				
AUC	.051	.191	-.020	.091	.493^**^	1			
DA	.114	.106	.040	.104	-.108	-.302	1		
MA	.086	.033	-.058	-.061	.643^**^	.692^**^	-.474^**^	1	
FA	.072	.012	-.062	-.084	.607^**^	.672^**^	-.581^**^	.991^**^	1

We observed a significant positive correlation of platelet aggregation and AUC for platelet aggregation with WHR (r = 0.307, p < 0.05), BMR (r = 0.356, p < 0.05), and FFM (0.363, p < 0.05). A significant positive correlation of age was observed with SBP and DBP. TBW was lower in obese compared to non-obese groups. In obese people, the percentage of people with no exercise was 30%, some exercise 19%, regular exercise 0%, and athlete 0%. In the non-obese group, the percentage of people with no exercise 11.9%, some exercise 23.8%, regular exercise 11.9%, and athlete 2.3%. The TBW was lower in the obese group compared to the non-obese group.

## Discussion

This study explored the relationship between body composition and cardiovascular risks in obese and non-obese individuals. There was a significant difference in most of the parameters of total body composition in obese and non-obese subjects. In contrast to the hypothesized association of increased adiposity and platelet function, no positive correlation was observed. However, the positive correlation between WHR and PA in all subjects in this study provides new insight into the role of WHR and the risk of enhanced platelet function. 

The role of adiposity in cardiovascular events has already been established [14]. Adiposity has been associated with thrombotic changes and evidence of increased coagulation and platelet activation. However, these changes did not seem to account for all the increased risk. Furthermore, there were different means of comparison between our study and the existing literature. For example, Farhangi et al. recruited 84 healthy women with a mean age of 35.56 +/- 6.83 years and they were categorized into two groups based on their BMI [15]. It has been shown that the platelet counts were also positively associated with WHR. This is consistent with our study showing a positive correlation between WHR and PA in all subjects.

Nonetheless, we observed that subjects with a positive family history of metabolic syndrome were indeed obese individuals according to the BMI classification. These findings are in line with the Santilli et al. study where they demonstrated that metabolic syndrome is a strong predictor of cardiovascular events regardless of BMI, thus suggesting a common downstream pathway conferring increased cardiovascular risk [16]. It conveyed that platelet hyperreactivity/activation plays a significant role in the accelerated thromboembolic incidents as a result of the interaction of the different inflammatory mediators involved in both obesity and metabolic syndrome.

In the current study, the blood pressure of obese subjects was significantly higher compared to the non-obese, which is in alignment with the earlier study by Cohen et al. [17]. The study conducted by Cohen et al. concluded that several interrelated mechanisms promote the development of hypertension in obesity, often contributing to end-organ damage, including cardiovascular disease. The findings from our current investigation revealed that obese individuals are at high risk of developing hypertension and increased platelet reactivity. However, such data could not be generalized due to some limitations of the study, including small sample size, confounding factors (diet and lifestyle), and the cross-sectional observational study design.

## Conclusions

We concluded that there was no direct significant correlation between adiposity and platelet activation in obese subjects. However, the study revealed a positive relationship between WHR and AUC with platelet aggregation. 
